# Varenicline decreases alcohol consumption in heavy-drinking smokers

**DOI:** 10.1007/s00213-012-2717-x

**Published:** 2012-05-01

**Authors:** Jennifer M. Mitchell, Candice H. Teague, Andrew S. Kayser, Selena E. Bartlett, Howard L. Fields

**Affiliations:** 1 Ernest Gallo Clinic and Research Center, University of California, San Francisco, 5858 Horton Street, Suite #200, Emeryville, CA 94608 USA; 2 Department of Neurology, University of California, San Francisco, Emeryville, CA 94143-0114 USA

**Keywords:** Chantix, Double-blind, Smoking, α4β2, Hazardous drinking, Abuse

## Abstract

**Rationale:**

Emerging evidence suggests that the α4β2 form of the nicotinic acetylcholine receptor (nAChR) modulates the rewarding effects of alcohol. The nAChR α4β2 subunit partial agonist varenicline (Chantix™), which is approved by the Food and Drug Administration for smoking cessation, also decreases ethanol consumption in rodents (Steensland et al., Proc Natl Acad Sci U S A 104:12518–12523, 2007) and in human laboratory and open-label studies (Fucito et al., Psychopharmacology (Berl) 215:655–663, 2011; McKee et al., Biol Psychiatry 66:185–190 2009).

**Objectives:**

We present a randomized, double-blind, 16-week study in heavy-drinking smokers (*n* = 64 randomized to treatment) who were seeking treatment for their smoking. The study was designed to determine the effects of varenicline on alcohol craving and consumption. Outcome measures included number of alcoholic drinks per week, cigarettes per week, amount of alcohol craving per week, cumulative cigarettes and alcoholic drinks consumed during the treatment period, number of abstinent days, and weekly percentage of positive ethyl glucuronide and cotinine screens.

**Results:**

Varenicline significantly decreases alcohol consumption (*χ* ^2^ = 35.32, *p* < 0.0001) in smokers. Although varenicline has previously been associated with suicidality and depression, side effects were low in this study and declined over time in the varenicline treatment group.

**Conclusions:**

Varenicline can produce a sustained decrease in alcohol consumption in individuals who also smoke. Further studies are warranted to assess varenicline efficacy in treatment-seeking alcohol abusers who do not smoke and to ascertain the relationship between varenicline effects on smoking and drinking.

## Introduction

Alcoholism has immense social, medical, and financial costs. Currently, three drugs are approved by the Food and Drug Administration (FDA) for the treatment of alcohol abuse and dependence in the USA: disulfiram, naltrexone, and acamprosate. While these compounds are effective, they frequently cause unpleasant side effects, compliance is generally low, and relapse following treatment is common (Bouza et al. 2004; Whitworth et al. 1996; Wright and Moore 1990), indicating a need for more effective therapeutic approaches.

Heavy alcohol and nicotine use commonly occur in the same individual (Toneatto et al. 1995). Smoking is more prevalent in heavy drinkers (Mello et al. 1987; Room 2004) and alcohol potentiates nicotine reward (Rose et al. 2004) and self-administration (Mitchell et al. 1995). Furthermore, animal studies indicate that neuronal nicotinic acetylcholine receptors (nAChRs), also contribute to the rewarding effects of alcohol (Bito-Onon et al. 2011). Importantly, varenicline, a partial agonist at α4β2 nAChRs, reduces both nicotine reward and ethanol seeking and consumption in rodent models (Chatterjee et al. 2011; Steensland et al. 2007). Consistent with these rodent studies, two recent human laboratory and open-label studies have reported that varenicline can reduce alcohol self-administration in heavy-drinking smokers (Fucito et al. 2011; McKee et al. 2009). These studies also suggest that varenicline is safe in humans who are using alcohol, an important factor in the development of new pharmacotherapies for individuals with alcohol abuse and/or dependence.

Despite the promising studies described above, the impetus to test varenicline in an alcohol-abusing population is tempered by reports that it exacerbates depression, hostility, and aggression (Moore et al. 2010) and the recent FDA ruling that varenicline carry a black box label for suicidal ideation and suicidal behavior (FDA Drug Safety Newsletter 2009). Although it remains vital to identify therapeutics that can be used in patients with psychiatric comorbidities, as these individuals comprise a significant portion of the heavy-drinking population (Regier et al. 1990; Tómasson and Vaglum 1995), concerns about possible adverse behavioral and emotional effects of varenicline in patients with psychiatric comorbidities made it imperative to carefully screen the present study cohort and to exclude subjects with a history of depression or suicidality.

Because subjective reporting can be unreliable in heavy drinkers (Heffernan et al. 2002; Toneatto et al. 1992), the use of objective markers of alcohol consumption provides important confirmatory data in studies of treatment efficacy. Recent advances in laboratory testing make it possible to measure long-lasting alcohol metabolites and biomarkers of alcohol consumption. Ethyl glucuronide (ETG) is a metabolite that is formed when ethanol interacts with glucuronic acid (Wurst et al. 1999) and is now commonly used to assay alcohol consumption. It is slowly eliminated from the urine over approximately 72 h (Schmitt et al. 1997) and is detectable in hair samples for many months (Appenzeller et al. 2007).

Here, we assessed the effects of varenicline on both alcohol and cigarette consumption and alcohol craving using electronic diaries, ETG testing, and a battery of behavioral questionnaires. We specifically tested the hypothesis that varenicline decreases craving and consumption of alcohol in nontreatment-seeking heavy drinkers who also smoke and that these effects would correlate with the effects of varenicline on cigarette consumption.

## Methods

### Subjects

Social drinkers (*n* = 99) were recruited online from Craigslist.org based on cigarette smoking (≥10 cigarettes/week) and alcohol consumption (≥7 drinks/week for women and ≥14 drinks/week for men). Subjects were invited to participate in a smoking cessation study. The words “alcoholic” and “alcohol abuse” were never used in conjunction with recruitment, screening, or data collection, and subjects were not treatment-seeking for alcohol abuse. Subjects were screened for physical dependence on alcohol and for psychiatric comorbidities and those that screened positive were excluded. During treatment, subjects were required to report daily alcohol and cigarette use online via online diaries on a secure study server. Subjects had until midnight to record the previous day’s substance use.

After obtaining written informed consent in accordance with the guidelines of the University of California, San Francisco, subject eligibility was assessed at a screening visit. This visit consisted of the administration of behavioral inventories: the Mini-International Neuropsychiatric Interview (Sheehan et al. 1998), the Alcohol Use Disorders Identification Test (AUDIT; Saunders et al. 1993), the Depression, Anxiety, and Stress Scale (DASS; Lovibond and Lovibond 1995), the Obsessive Compulsive Drinking Scale (OCDS; Anton et al. 1996), the Barratt Impulsivity Scale (Patton et al. 1995), and the Fagerstrom Test for nicotine dependence (Heatherton et al. 1991). The screening visit also included a physical examination, a 12-lead electrocardiogram (ECG), and a blood draw to determine liver function. The study physician used the Diagnostic and Statistical Manual of Mental Disorders, Fourth Edition criteria to assess alcohol dependence. Alcohol-dependent subjects were excluded. During the screening visit, subjects were asked if they were currently or had ever been treatment-seeking for alcohol abuse or dependence, and those that reported in the affirmative were excluded from the study. At all visits, subjects were screened for illicit drug use (amphetamine, methamphetamine, cocaine, opioids, and tetrahydrocannabinol: Quiktest, Voorhees, NJ, USA; phencyclidine and benzodiazepines at screening only: Biotechnostix, Ontario, Canada), for cotinine (Innovacon, San Diego, CA, USA), and for alcohol intoxication using a breathalyzer (Lifeloc Technologies, Wheat Ridge, CO, USA). Female subjects were administered a pregnancy test (at screening and then monthly; Earth’s Magic, Morrisville, NC, USA). Subjects were also administered the DASS, the OCDS, the Fagerstrom, and a Likert side effects scale (four-ordered response levels, where 0 = not at all and 3 = severe) with 38 questions. Inclusion criteria included a score of >8 on the AUDIT, indicating hazardous drinking (or a score of >4 when a staff member administered 3 questions from the AUDIT as a check of the participant’s self-report), a score of <32 on the DASS, no risk of pregnancy, a BAL <0.05 (percent per volume) to consent, and no more than twice weekly use of illicit substances. Eligible subjects (*n* = 64) were then randomized into one of two treatment groups in a double-blind fashion. Thirty-five subjects completed the 12-week medication cycle and 34 subjects completed the study through week 16. Diary data from one subject was dropped from data analysis because he told study staff that he had shared his username and password with his girlfriend so that she could monitor his alcohol intake and implied that his diaries were, therefore, inaccurate. Study visits took place at the Ernest Gallo Clinic and Research Center in Emeryville, CA, USA. Subjects had 24-h phone access to the study nurse practitioner. All subjects were paid for their participation ($20 per visit and $80 for full study completion = $400 maximum payment for the 16-week study).

**Fig. 1:**
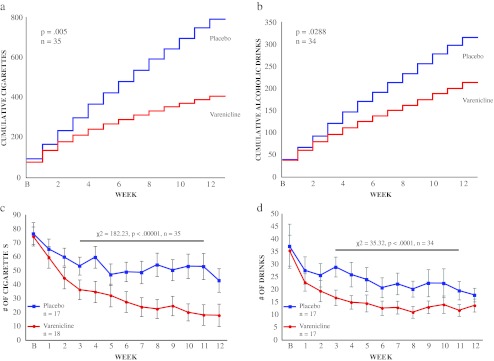
Varenicline effects on smoking and alcohol consumption. Varenicline significantly decreases **a** cumulative cigarette consumption (*p* = 0.005, *n* = 35) and **b** cumulative alcohol consumption (*p* = 0.0288, *n* = 34). **c** Varenicline significantly reduced average number of cigarettes smoked from weeks 3 to 11 (*χ* ^2^ = 182.23, *p* < .00001). **d** Varenicline significantly reduced average number of drinks consumed from weeks 3 to 11 (*χ* ^2^ = 35.32, *p* < .0001)

### Study design

This was an outpatient, double-blind, placebo-controlled study. Eligible subjects were randomized to either varenicline (1 mg twice daily, *n* = 29) or placebo (*n* = 35) twice daily for 12 weeks (the recommended period for varenicline treatment of nicotine dependence). The 12-week treatment period included drug titration at onset (0.5 mg once daily for 3 days, followed by 0.5 mg twice daily for 4 days) and offset (0.5 mg twice daily for 2 days, 0.5 mg once daily for 2 days) to mitigate side effects. Two follow-up visits were conducted at weeks 14 and 16. Participants were encouraged to quit smoking 1 week into study drug treatment and were offered weekly group therapy sessions to aid in this endeavor. Three subjects participated in the therapy sessions (one time each). As our research question addressed whether alcohol drinking is attenuated by varenicline treatment, only nontreatment-seeking heavy drinkers were included in this study and no effort was made to discourage their use of alcohol.

### Computerized alcohol use diaries

Subjects were given a coded username and password to log on to the study server where they entered the number of alcoholic drinks and cigarettes they consumed during each 24-h period. Subjects had until midnight to record the previous day’s drinking and smoking. Standard drinks and alcohol content were calculated from the information provided. Subjects also used their electronic diaries to report medication use, to report any nonstudy-related illness, to keep notes on side effects, and to report anything else they felt might be of interest to the experimenters. Subjects with a full set of diary data received a monetary bonus at the end of the study.

### Study drug

Study drug was provided by Pfizer pharmaceuticals and was reconstituted (to add riboflavin and to match placebo in appearance) and blinded by Abbotts Compounding Pharmacy in Berkeley, CA, USA. Study drug was dispensed by the clinic pharmacist, who also randomized study drug allocation. Subjects were instructed to take their medication twice daily. Riboflavin (25 mg), which causes urine samples to fluoresce under ultraviolet (UV) light, was added to both drug and placebo capsules to monitor drug compliance. Urine samples were collected weekly. Additionally, medication bottles were all equipped with Medication Event Monitoring System caps (MEMS caps; AARDEX, Zurich, Switzerland), which recorded the time of each cap removal, and subjects were instructed to only open their medication bottles to take their study drug.

### Diagnostic testing

ETG testing was conducted weekly by Phamatech Laboratories (San Diego, CA, USA). Complete blood counts and hepatic function panels were performed by Quest Diagnostics.

### Data analysis

The primary outcome measures were calculated as both alcoholic drinks per week and cigarettes per week, as well as alcohol craving per week (OCDS). Secondary outcome measures included cumulative cigarettes and alcoholic drinks consumed during the treatment period, number of days abstinent, and weekly percentage of positive ETG and cotinine screens. Data for primary outcome measures were analyzed in two separate ways: (1) using all subjects randomized to treatment and (2) using subjects that completed all 12 weeks of study drug. Cumulative consumption curves were plotted for varenicline and placebo completers and the point of bifurcation (week 3) was taken as indicative of the onset of a treatment effect and, therefore, as the first analysis point for chi square. The initiation of taper down (week 11) was taken as the final data point. Individual change scores (where pretreatment = baseline data and posttreatment = week 12 data) for smoking and drinking were calculated for each subject completing the study and were compared using regression analysis. Pearson’s correlation analysis, regression analysis, and chi square were conducted using Excel and SPSS. Differences were considered significant if *p* < 0.05. Permutation analysis (1,000,000 shuffles to generate the null distribution) was conducted with MatLab. The mean of the differences of the observed data were compared to the null distribution of the permutated differences and considered significant if *p* < 0.05 (one-tailed). Analytical methods were chosen and reviewed in consultation with the UCSF Biostatistical Core.

## Results

### Demographics and retention

Drug cycle populations were demographically well-matched for variables including gender, education, ethnicity, and age (Table 1). There was no significant difference in rate of retention between the varenicline and placebo control groups over the 12-week treatment period (*χ* ^2^ = 0.688, *p* = 0.61). At the end of the 12-week treatment period, dropout rate was 45.17 % for the varenicline treatment group and 35.72 % for the placebo group. The most common explanations given for termination of study participation were time constraints, upcoming travel, and change in job or living arrangement. Five drop outs were due to adverse events (AEs; see below).

**Table 1:** Patient demographic data

	Placebo, *n* = 31	Varenicline, *n* = 33
Age in years		
Median	25	29
Minimum/maximum	21:44	21:59
Sex, *n* (%)		
Women	11 (35)	15 (45)
Men	20 (65)	18 (55)
Baseline drinks/week		
Mean	37	35
Minimum/maximum	5–144	3–105
Baseline cigarettes/week		
Mean	77	75
Minimum/maximum	16–183	26–175
Baseline inventories		
AUDIT score	19.46	16.16
OCDS score	12.0	11.0
Fagerstrom score	3.32	3.68
Race/ethnicity, *n* (%)		
African American	3 (10)	4 (12)
Caucasian	20 (64)	24 (73)
Mixed	4 (13)	1 (3)
Hispanic	3 (10)	1 (3)
Asian	1 (3)	1 (3)
Native American	0 (0)	1 (3)
No identification	0 (0)	1 (3)
Education level, *n* (%)		
High school	3 (10)	4 (12)
Associates degree	2 (6)	1 (3)
Trade school	2 (6)	2 (6)
Some college	17 (55)	12 (36)
Bachelors degree	5 (16)	12 (36)
Graduate school	2 (6)	2 (6)
AEs, *n* (%)	1 (3)	3 (9)

### Electronic diaries

Online diary drinking data were positively correlated with AUDIT score, indicating that our online diary data are reasonable indicators of problematic alcohol use (*R* = 0.55, *p* < 0.00001, *n* = 58). Online diary drinking data also positively correlated with weekly OCDS scores over the duration of study drug treatment (*R* = 0.93, *p* ≤ 0.00001), indicating a tight association between craving for alcohol and alcohol consumption within the same period of time.

### Cigarette consumption

Since cumulative smoking diverged for varenicline and placebo treatment groups at week 3 (Fig. 1a), chi square analysis was conducted on smoking data from weeks 3 to 11 (when taper down was initiated). In keeping with previous reports, there was a significant difference in cigarettes per week for completers in the varenicline group versus the placebo group from weeks 3 to 11 (*χ* ^2^ = 182.23, *p* < 0.00001; Fig. 1c). Permutation analysis revealed a significant difference in cumulative cigarettes smoked between the varenicline and placebo groups for completers (*p* = 0.005, *n* = 35; mean placebo group cigarettes = 788.94, mean varenicline group cigarettes = 403.24). Similarly, there was a significant difference in cigarettes per week for all subjects randomized to treatment in the varenicline group versus the placebo group from weeks 3 to 11(*χ* ^2^ = 137.47, *p* < 0.00001), as well as a significant difference in cumulative cigarettes smoked between the varenicline and placebo groups for all subjects randomized to treatment (*p* = 0.019, *n* = 58; mean placebo group cigarettes = 479.43, mean varenicline group cigarettes = 272.97).

### Alcohol consumption

Since cumulative drinking also diverged for varenicline and placebo treatment groups at week 3 (Fig. 1b), chi square analysis was conducted on drinking data from weeks 3 to 11 (when taper down was initiated). There was a significant difference in drinks per week between completers in the varenicline and placebo groups from weeks 3 to 11 (*χ* ^2^ = 35.32, *p* < 0.0001, *n* = 34; Fig. 1d). Permutation analysis revealed a significant difference in cumulative drinks between the varenicline and placebo control groups for completers (*p* = 0.029, *n* = 34; mean placebo drinks = 277.5 ± 41.9, mean varenicline drinks = 177.04 ± 28.86). There was also a significant difference in drinks per week in the varenicline and placebo groups from weeks 3 to 11 in all subjects randomized to treatment (*χ* ^2^ = 26.34, *p* < 0.0001, *n* = 58), as well as a significant difference in cumulative drinks between the varenicline and placebo control groups for all subjects randomized to treatment (*p* = 0.017, *n* = 58; mean placebo = 224.12 ± 28.9, mean varenicline drinks = 144.60 ± 20.56).

### Alcohol craving

Chi square analysis conducted on craving data from weeks 3 to 11 indicated a trend toward significance between groups for completers, such that varenicline-treated subjects reported less craving (*χ* ^2^ = 13.53, *p* < 0.09, *n* = 35). There was no significant difference between groups in all subjects randomized to treatment (*χ* ^2^ = 12.03, *p* = 0.14, *n* = 58).

### Nicotine dependence

Surprisingly, we did not replicate the previous report that varenicline significantly attenuates nicotine dependence in either completers (*χ* ^2^ = 1.964, *p* = 0.982, *n* = 35) or all subjects randomized to treatment (*χ* ^2^ = 1.4936, *p* = 0.992, *n* = 58).

### Metabolic measures of consumption

Consistent with patient reports, there was a significant difference in ETG-negative subjects in the varenicline treatment group versus the placebo treatment group at week 12 for completers (placebo = 33.3 % negative, varenicline = 53.0 % negative, *χ* ^2^ = 11.74, *p* < 0.001, *n* = 35), but no significant difference between percentage of cotinine-negative subjects in the varenicline treatment group versus the placebo treatment group at week 12 for completers (*χ* ^2^ = 0.008, *p* = 0.93, *n* = 35).

### Relationship between smoking and drinking

There was no correlation between average number of drinks consumed per week and average number of cigarettes smoked per week when individual change scores were calculated for each subject, indicating that, while varenicline influenced both alcohol and cigarette consumption, these values were not always identically affected by varenicline treatment.

### Abstinence

Varenicline did not affect the number of days completers reported being abstinent from alcohol, suggesting that, during varenicline treatment, subjects maintained their pattern of initiating alcohol consumption, but drank less once drinking was initiated. This abstinence-independent change in consumption may reflect a change in the reward value of alcohol, as varenicline also effectively reduces alcohol intake in a single-session laboratory bar paradigm (McKee et al. 2009), consistent with the hypothesis that it reduces the incentive to continue drinking once drinking is initiated.

### Side effects

Although varenicline carries a black box warning for “hostility, agitation, depressed mood, and suicide related events, including ideation, behavior, and attempted suicide,” subjects generally reported a low rate of side effects and these were not significantly different between groups (mean placebo group at baseline = 10.36 ± 1.22 versus mean varenicline group at baseline = 8.39 ± 1.58; mean placebo group at week 12 = 2.67 ± 0.89 versus mean varenicline group at week 12 = 1.41 ± 0.83). DASS scores were also low (mean placebo group at baseline = 13.68 ± 1.99 versus mean varenicline group at baseline = 8.13 ± 1.56; mean placebo group at week 12 = 1.56 ± 0.63 versus mean varenicline group at week 12 = 1.06 ± 0.63) throughout the study. Additionally, a one-way repeated-measures analysis of variance indicated that side effects decreased significantly over time in all subjects (*F* = 7.51, *p* < 0.00001, *n* = 13). While such improvements are typical in studies that involve constant monitoring and care of a clinical population, these data may also reflect the rigorous exclusion of subjects with comorbid disorders.

### Adverse events

An AE was defined as either a “severe” rating on any of the side effects scale questions or an unanticipated study termination due to side effects. Five AEs were reported during this study: (1) a 30-year-old female reported anger, aggression, and vivid nightmares, (2) a 23-year-old female reported nausea and vomiting, (3) a 25-year-old female reported headache and nausea, (4) a 42-year-old male reported anger, agitation, and sleep disturbances, and (5) a 21-year-old male reported suicidal thinking. Of these five AEs, the first four occurred while subjects were taking varenicline and the last AE occurred on placebo. All subjects that experienced an AE in the varenicline group were also positive for psychostimulants at their last study visit.

ECG data obtained at baseline and week 12 did not indicate any effect of drug treatment on cardiovascular function.

### Compliance

MEMS cap data indicated that subjects opened their medication bottles at least once per day an average of 6.2 days/week. There was no significant difference in the number of bottle openings between placebo and varenicline subjects. Urine samples of all subjects tested positive for riboflavin, as measured by visual assessment under UV light, indicating a high level of compliance with the study drug schedule.

## Discussion

We find that varenicline attenuates alcohol consumption in smokers. Varenicline also reduces reported cumulative consumption of alcohol and ongoing consumption, as measured using the objective marker ETG. Importantly, in view of the well-established efficacy of varenicline to reduce smoking, the effect on drinking uncovered in this study supports the hypothesis that varenicline will have clinical benefit for heavy-drinking smokers. Furthermore, it is consistent with animal studies indicating that alcohol and nicotine act through a common reward pathway that involves the NAChR (Bito-Onon et al. 2011; Chatterjee et al. 2011; Hendrickson et al. 2010; Steensland et al. 2007). Although our data indicate that the varenicline-induced reduction of alcohol consumption is independent of the effects of varenicline on smoking, additional studies must be conducted in nonsmoking drinkers to definitively determine whether varenicline would be effective in individuals who do not smoke.

Subjects were told that this was a smoking cessation study and, as such, were asked to identify a quit date. However, subjects were not excluded from the study if they failed to adhere to the quit date or if they chose not to attend the offered group therapy for smoking cessation, mitigating the pressure to actually quit smoking. Only three subjects participated in the offered group therapy sessions and only one subject adhered to their self-ascribed quit date, complicating the possibility of analysis based on smoking cessation. If more subjects had actually quit smoking, it might have been interesting to compare drinking between quitters and nonquitters. In the absence of this comparison, it is not possible to determine how much of the effect of varenicline on drinking was secondary to its effect on smoking.

In light of previously reported findings indicating the effectiveness of open-label varenicline in reducing heavy alcohol consumption among a treatment-seeking population (Fucito et al. 2011), it was important here to assess the effects of varenicline on drinking in subjects who were not treatment-seeking for alcohol abuse and who were not self-described “alcoholics.” The use of a nontreatment-seeking population can control for sample bias and regression to the mean, as subjects are not explicitly attempting to attenuate their drinking and, therefore, are not necessarily expecting to drink less during the course of drug therapy. Our use of nontreatment-seeking drinkers also allowed us to isolate the effects of drug treatment without influence from other psychosocial strategies, such as Alcoholics Anonymous, group therapy, family support, etc. that treatment seekers may simultaneously pursue in an effort to decrease their intake. Additionally, from a public health perspective, it is important to identify treatments that can be used to effectively reduce drinking independent of abstinence. However, given that the present subject population was seeking treatment for smoking cessation, it remains possible that decreased alcohol craving and consumption was due in part to an attempt to quit smoking and to avoid places, such as bars, where smoking and drinking commonly occur together.

As in many such study populations, subject retention was problematic (Howard 1992) and our attrition rate was magnified by the long study duration (16 weeks). Dropouts occurred with equal frequency in both groups, and there were no significant demographic differences between those subjects who failed to complete the 16-week study and completers. Subjects’ own reasons for leaving the study were rarely related to concerns about the efficacy, or lack thereof, of the study drug. Thus, we have no reason to believe that attrition biased our results.

Varenicline carries a black box warning for suicidal ideation and suicidal behavior and has also been reported to exacerbate depression, hostility, and aggression (Moore et al. 2010; but see also Garza et al. 2011). Though anecdotal, it is of interest that all four of the AEs that occurred in subjects on active study drug were associated with concurrent psychostimulant use. It is possible that some subjects’ use of cocaine and methamphetamine during the study exacerbated the potential for varenicline to induce these negative and potentially dangerous side effects. Although a previous study suggested that varenicline side effects are not exacerbated in cocaine users (Poling et al. 2010), our results suggest that care should be taken when administering varenicline to alcohol abusers who are concurrently using psychostimulants. However, further studies are necessary to definitively assess the contribution of psychostimulants to varenicline AEs.

California has one of the smallest smoking populations in the USA (http://www.dhs.ca.gov/tobacco) and, while identification of smokers seeking cessation treatment was not difficult in our population, it was uncommon to encounter an individual smoking a half a pack or more per week. Additionally, our cotinine results suggest that, although subjects were smoking significantly less at the end of the 12 weeks of varenicline treatment, they were, by and large, still smoking. Though our data indicates lower efficacy than previous reports on varenicline treatment of smoking (Gonzales et al. 2006; Jorenby et al. 2006), these data may also reflect our moderate inclusion criteria: subjects were asked to plan a quit date but were not dropped from the study if they did not meet this quit date nor were they dropped if they relapsed to cigarette smoking during treatment.

## Conclusion

We find that varenicline (Chantix™) is effective at inhibiting not only cigarette smoking, as previously reported, but also inhibits alcohol consumption in a population of heavy drinkers who were not seeking treatment for their drinking. Although adverse side effects were minimal, further clinical studies are necessary to determine whether varenicline is equally safe in study populations with comorbid psychiatric conditions as well as in nonsmoking alcohol-dependent individuals.
